# Plasma Biomarkers and Clinical Outcomes in Early-Onset Dementia

**DOI:** 10.1001/jamanetworkopen.2026.9687

**Published:** 2026-04-29

**Authors:** Hyemin Jang, Sun Min Lee, Hee Jin Kim, Na-Yeon Jung, Henrik Zetterberg, Kaj Blennow, Liana G. Apostolova, So Young Moon, Eun-Joo Kim, and the Longitudinal Study of Early-Onset Dementia and Family Members (LEAF) Investigators

**Affiliations:** 1 Department of Neurology, Asan Medical Center, University of Ulsan College of Medicine, Seoul, Korea; 2 Department of Neurology, Ajou University School of Medicine, Suwon, Korea; 3 Department of Neurology, Samsung Medical Center, Sungkyunkwan University School of Medicine, Seoul, Korea; 4 Department of Neurology, Pusan National University Yangsan Hospital, Research Institute for Convergence of Biomedical Science and Technology, Yangsan, Korea; 5 Department of Psychiatry and Neurochemistry, Institute of Neuroscience and Physiology, the Sahlgrenska Academy at the University of Gothenburg, Gothenburg, Sweden; 6 Clinical Neurochemistry Laboratory, Sahlgrenska University Hospital, Gothenburg, Sweden; 7 Department of Neurodegenerative Disease, University College London (UCL) Institute of Neurology, Queen Square, London, United Kingdom; 8 UK Dementia Research Institute at UCL, London, United Kingdom; 9 Hong Kong Center for Neurodegenerative Diseases, Clear Water Bay, Hong Kong, China; 10 Wisconsin Alzheimer’s Disease Research Center, University of Wisconsin School of Medicine and Public Health, University of Wisconsin-Madison, Madison; 11 Department of Pathology and Laboratory Medicine, University of Wisconsin School of Medicine and Public Health, University of Wisconsin-Madison, Madison; 12 Centre for Brain Research, Indian Institute of Science, Bangalore, India; 13 Neurodegenerative Disorder Research Center, Division of Life Sciences and Medicine, and Department of Neurology, Institute on Aging and Brain Disorders, University of Science and Technology of China (USTC) and First Affiliated Hospital of USTC, Hefei, China; 14 Indiana Alzheimer’s Disease Research Center, Indianapolis, Indiana; 15 Department of Neurology, Indiana University School of Medicine, Indianapolis; 16 Department of Medical and Molecular Genetics, Indiana University School of Medicine, Indianapolis; 17 Department of Radiology and Imaging Sciences, Center for Neuroimaging, Indiana University School of Medicine Indianapolis, Indianapolis; 18 Department of Neurology, Pusan National University Hospital, Pusan National University School of Medicine and Medical Research Institute, Busan, Korea

## Abstract

**Importance:**

Clinical utility and dynamics of plasma biomarkers in early-onset dementia remain underexplored.

**Objective:**

To investigate plasma biomarker trajectories and their associations with clinical outcomes in early-onset Alzheimer disease (EOAD) and frontotemporal dementia (FTD).

**Design, Setting, and Participants:**

This multicenter, prospective cohort study analyzed participants in phase 1 of the Longitudinal Study of Early-onset Dementia and Family Members (LEAF), which was conducted from April 2021 through December 2023 in 34 centers across South Korea. Patients with β-amyloid–positive EOAD and FTD were included and underwent annual blood sampling and clinical assessment, within a follow-up period of approximately 2 years. Data were analyzed between June 2025 and March 2026.

**Exposure:**

Levels of plasma phosphorylated tau 217 (p-tau217), glial fibrillary acidic protein (GFAP), and neurofilament light chain (NfL) biomarkers were analyzed using assays.

**Main Outcomes and Measures:**

(1) Associations of baseline biomarkers with clinical outcomes (assessed with the Mini-Mental State Examination [MMSE] and the Clinical Dementia Rating–Sum of Boxes [CDR-SB] for the EOAD group, or the frontotemporal lobar degeneration [FTLD]–modified CDR-SB for the FTD group), (2) biomarker trajectories, and (3) association of biomarker level changes and clinical outcomes.

**Results:**

A total of 322 participants with p-tau217, GFAP, and NfL analyses were stratified into the EOAD or FTD group based on their diagnosis. The EOAD group (n = 245) had a mean (SD) age of 61.8 (5.4) years and included 163 females (66.5%), while the FTD group (n = 77) had a mean (SD) age of 65.1 (7.3) years and included 45 females (62.3%). In the EOAD group, higher log_2_-transformed baseline p-tau217, GFAP, and NfL were each associated with faster decline in the MMSE score (association estimate [SE], –0.390 [0.127], *P* = .002; –0.775 [0.164], *P* < .001; and –0.679 [0.182], *P* < .001, respectively) and the CDR-SB score (estimate [SE], 0.401 [0.099], *P* < .001; 0.535 [0.126], *P* < .001; and 0.693 [0.122], *P* < .001, respectively). In the FTD group, GFAP and NfL were associated with MMSE decline (estimate [SE], –2.118 [0.566], *P* < .001 and –2.360 [0.428], *P* < .001, respectively), whereas p-tau217 was not (estimate [SE], 0.071 [0.418], *P* = .87). No biomarker was associated with FTLD-modified CDR-SB score change. Longitudinally, all mean (SD) biomarker levels increased in the EOAD group (p-tau217: 0.253 [0.077] pg/mL, *P* = .001; GFAP: 0.173 [0.040] pg/mL, *P* < .001; NfL: 0.149 [0.045] pg/mL, *P* = .001), whereas in the FTD group, only NfL level showed an upward pattern (0.251 [0.127] pg/mL, *P* = .05). Annualized biomarker changes were associated with worsening clinical outcomes in the EOAD group, but not in the FTD group. GFAP and NfL level increases were associated with MMSE score decline (estimate [SE], –0.005 [0.002], *P* = .007 and –0.010 [0.003], *P* = .001, respectively), while p-tau217 level increases were associated with CDR-SB score worsening (estimate [SE], 0.072 [0.024], *P* = .003) in the EOAD group.

**Conclusions and Relevance:**

In this cohort study of patients with EOAD and FTD, baseline p-tau217, GFAP, and NfL were consistently associated with clinical outcomes in the EOAD group, whereas GFAP and NfL were associated with cognition only in the FTD group. These findings demonstrate distinct characteristics of plasma biomarkers in EOAD and FTD, supporting their potential utility for risk stratification.

## Key Points

**Question:**

Are plasma phosphorylated tau217 (p-tau217), glial fibrillary acidic protein (GFAP), and neurofilament light chain (NfL) biomarkers and their longitudinal changes associated with clinical outcomes in early-onset Alzheimer disease (EOAD) and frontotemporal dementia (FTD)?

**Findings:**

In this cohort study of 322 patients with EOAD and FTD, all biomarkers were associated with decline in the Mini-Mental State Examination (MMSE) and Clinical Dementia Rating–Sum of Boxes (CDR-SB) scores in the EOAD group; in the FTD group, only GFAP and NfL were associated with MMSE score decline. In the EOAD group, annualized p-tau217 level increases were associated with CDR-SB score worsening, whereas GFAP and NfL level increases were associated with MMSE decline; no such associations were observed in the FTD group.

**Meaning:**

The findings suggest that plasma biomarkers’ distinct trajectories and clinical associations in EOAD and FTD support their potential utility for risk stratification.

## Teaser

This cohort study evaluates the association of long-term changes in phosphorylated tau217, glial fibrillary acidic protein, and neurofilament light chain biomarkers with clinical outcomes in individuals with early-onset Alzheimer disease and frontotemporal dementia.

## Introduction

Early-onset Alzheimer disease (EOAD) and frontotemporal dementia (FTD) are the leading causes of early-onset dementia or young-onset dementia, which manifests symptoms before the age of 65 years.^1^ While EOAD shares core pathology with late-onset AD, it may present distinct clinical, genetic, and biomarker features.^2,3,4,5^ FTD, although also seen in later life, is a major contributor to early-onset dementia. Diagnosing early-onset dementia is challenging due to atypical symptoms and clinical heterogeneity, which complicates both early detection and prognostication. To address these gaps, the Longitudinal Study of Early-onset Dementia and Family Members (LEAF) cohort was initiated in South Korea in 2021, aiming to characterize early-onset dementia in terms of longitudinal clinical features, risk factors, pathogenic variants, and biomarkers.

Recent developments in plasma biomarkers have changed the landscape of dementia diagnosis. Phosphorylated tau 217 (p-tau217), an AD-specific marker, has demonstrated high accuracy in detecting AD pathology.^6^ Glial fibrillary acidic protein (GFAP) and neurofilament light chain (NfL) are emerging as astrocytic activation and neurodegeneration markers, respectively,^7,8,9^ with NfL particularly relevant for FTD.^10,11^ Thus, combining p-tau217 and NfL may enable differential diagnosis between AD and FTD.^12,13^ In addition to their diagnostic potential, numerous studies, including a large-scale Korean study, have shown associations between these biomarkers and cognitive decline.^12^ However, most prior investigations did not specifically focus on EOAD, thereby limiting the generalizability in this population. One study observed more elevation of NfL and GFAP in EOAD compared with late-onset AD, possibly reflecting the more aggressive clinical trajectory typically observed in EOAD.^14^ These findings imply that the diagnostic and prognostic performance of plasma biomarkers may be distinct in EOAD, emphasizing the need for studies specifically targeting this population. Furthermore, few studies have systematically examined the longitudinal changes in biomarkers associated with cognitive decline in sporadic EOAD, and this is equally true for FTD, underscoring the need for further research in these clinically distinct groups.

In this study, we aimed to investigate biomarker (plasma p-tau217, GFAP, and NfL) trajectories and their associations with clinical outcomes in EOAD and FTD subgroups from the LEAF cohort. Specifically, we examined (1) how p-tau217, GFAP, and NfL changed over time in EOAD and FTD, and (2) whether baseline levels or longitudinal changes in these biomarkers were associated with cognitive and functional decline in EOAD and FTD. We hypothesized that baseline levels and longitudinal changes in plasma biomarkers would show differential associations with clinical outcomes in EOAD vs FTD, supporting their distinct utility for disease monitoring and trial stratification.

## Methods

### Study Participants

The LEAF study is an observational clinical cohort involving 34 centers across South Korea and has been conducted in 2 consecutively funded phases under an identical protocol with continuous follow-up. LEAF1 was conducted from April 2021 through December 2023, while LEAF2 began in March 2024 and is planned to continue through December 2026.^15,16^ The present study included only participants enrolled during the LEAF1 phase. The institutional review board of each of the 34 centers approved this prospective cohort study. All participants provided written informed consent. Study data were collected in accordance with the Declaration of Helsinki.^17^ We followed the Strengthening the Reporting of Observational Studies in Epidemiology (STROBE) reporting guideline.

At baseline, all participants underwent physical and neurological examinations, neuropsychological tests, detailed questionnaires, blood sampling, brain magnetic resonance imaging, and β-amyloid (Aβ) testing either through Aβ-positron emission tomography scans (eMethods in Supplement 1) or cerebrospinal fluid testing. Thus, the LEAF cohort included patients with Aβ-positive EOAD continuum, FTD, and other forms of early-onset dementia. The EOAD continuum encompassed subjective cognitive decline, mild cognitive impairment, and AD dementia, as defined by their respective diagnostic criteria.^18,19,20^ FTD syndrome included 6 clinical subtypes defined by applicable clinical criteria: behavioral variant FTD, semantic variant primary progressive aphasia, nonfluent/agrammatic variant primary progressive aphasia, FTD-motor neuron disease, progressive supranuclear palsy syndrome, and corticobasal syndrome.^21,22,23,24,25^

### Plasma Collection and Processing

Blood was collected in tubes containing 0.5-M EDTA, centrifuged, and separated into 0.3-mL vial before storage at −80 °C. Plasma samples were sent for analysis to the Department of Psychiatry and Neurochemistry at the University of Gothenburg. The in-house p-tau217 assay (University of Gothenburg p-tau217) was used on an immunoassay platform (Simoa HD-X; Quanterix).^26^ Plasma GFAP and NfL were measured using an assay kit (Neurology 4-Plex E; Quanterix). Details are provided in eMethods in Supplement 1.

### Longitudinal Plasma Biomarker and Cognitive and Functional Outcomes Assessment

In the LEAF cohort, participants underwent annual follow-up visits, including clinical assessment and blood sampling. In this study, follow-up plasma biomarker data were available for 131 participants with EOAD and 37 participants with FTD, with a median (IQR) sampling interval of 0.98 (0.92-1.06) and 1.01 (0.94-1.08) years, respectively. Cognitive and functional follow-up was conducted in the EOAD group using the Mini-Mental State Examination (MMSE; score range: 0-30, with higher scores indicating better cognitive function) and the Clinical Dementia Rating–Sum of Boxes (CDR-SB; score range: 0-30, with higher scores indicating greater cognitive and functional impairment). In the FTD group, follow-up was assessed using the MMSE and the frontotemporal lobar degeneration (FTLD)–modified CDR-SB (score range: 0-24, with higher scores indicating greater cognitive and functional impairment).^27,28^ In the EOAD group, the median (IQR) number of visits was 2.0 (2.0-3.0) and a median (IQR) follow-up of 2.0 (1.0-2.9) years. Participants in the FTD group had a median (IQR) of 2.0 (1.0-3.0) visits and a median (IQR) follow-up of 1.48 (1.1-2.5) years.

### Statistical Analysis

Plasma biomarker values below the lower limit of quantification were imputed as one-half of the lowest observed value. Continuous variables were summarized as mean (SD) and categorical variables as number (percentage). Group differences were assessed using independent 2-sample *t* tests or χ^2^ tests, as appropriate. The index date was defined as the first plasma sampling date, and baseline cognitive and functional measures were the assessments closest to this date. Longitudinal outcomes were aligned to the index date using all available follow-up data. Plasma biomarker concentrations were log_2_-transformed to reduce skewness.

Linear mixed-effects models were used to evaluate (1) associations between baseline biomarker levels and longitudinal changes in cognitive and functional outcomes, (2) longitudinal changes in plasma biomarker levels, and (3) associations between annualized biomarker level changes and clinical decline. Because absolute and proportional changes represent different effect scales, annualized biomarker change was parameterized on the raw scale (absolute change) with the log_2_-scale (proportional change) in sensitivity analyses. Models included time, biomarker terms, and their interactions as the fixed effects, with age, sex, educational level, and apolipoprotein E (*APOE*) ε4 genotype (EOAD group only) as the covariates. Participants were included as random intercepts. Standardized β coefficients were calculated to facilitate the comparison of effect sizes across outcomes.

Two-sided *P* < .05 indicated statistical significance. All statistical analyses were conducted between June 2025 and March 2026 using R, version 4.4.1 (R Project for Statistical Computing). Details are provided in the eMethods in Supplement 1.

## Results

### Characteristics of Study Participants

A total of 410 participants were enrolled in the LEAF1 cohort, of whom 322 were stratified into the EOAD or FTD group and received p-tau217, GFAP, and NfL analysis. The EOAD group included 245 participants (mean [SD] age, 61.8 [5.4] years; 163 females [66.5%] and 82 males [33.5%]); 2 participants had subjective cognitive decline, 46 had mild cognitive impairment, and 197 had AD dementia, among whom 128 (52.2%) were carrying *APOE* ε4. These patients had mean (SD) MMSE and CDR-SB scores of 18.0 (6.8) and 6.5 (5.1), respectively. The FTD group included 77 participants (mean [SD] age, of 65.1 [7.3] years; 45 females [62.3%] and 32 males [37.7%]. Of these participants, 32 had behavioral variant FTD, 29 had semantic variant primary progressive aphasia, 11 had nonfluent/agrammatic variant primary progressive aphasia, 3 had corticobasal syndrome, and 2 had progressive supranuclear palsy syndrome. These patients had mean (SD) MMSE and FTLD-modified CDR-SB scores of 17.3 (8.7) and 8.9 (6.2), respectively.

At baseline, the mean (SD) plasma p-tau217 and GFAP levels were higher in the EOAD group than in the FTD group (7.7 [4.1] and 202.0 [88.8] pg/mL vs 3.4 [2.4] and 127.0 [78.6] pg/mL). The mean (SD) NfL levels were higher in the FTD group compared with the EOAD group (57.4 [43.1] pg/mL vs 29.7 [20.2] pg/mL) (Table 1). We compared baseline characteristics between participants with and without clinical follow-up. In the EOAD group, those lost to follow-up had worse baseline median (IQR) MMSE scores (16.0 [9.0-22.0] vs 19.0 [16.0-24.0]; *P* = .003) and higher median (IQR) p-tau217 levels (9.5 [5.7-11.8] vs 6.9 [5.1-9.1]; *P* = .02). In the FTD group, those lost to follow-up had higher median (IQR) NfL levels (60.9 [42.1-141.0] vs 40.5 [25.6-55.6]; *P* = .002) (eTable 1 in Supplement 1).

**Table 1.** Baseline Demographic and Clinical Characteristics of Patients

Characteristic	Patients, mean (SD)		
	EOAD group (n = 245)	FTD group (n = 77)	*P* value
Age, y	61.8 (5.4)	65.1 (7.3)	<.001
Sex, No. (%)			
Male	82 (33.5)	32 (37.7)	.59
Female	163 (66.5)	45 (62.3)	
Educational level, y	11.8 (3.8)	10.6 (4.6)	.04
Onset to diagnosis, y	5.1 (3.0)	4.7 (3.2)	.26
*APOE* ε4 carrier status, No. (%)	128 (52.2)	12 (15.6)	<.001
MMSE score	18.0 (6.8)	17.3 (8.7)	.48
CDR-SB score	6.5 (5.1)	6.7 (5.2)	.76
FTLD-modified CDR-SB score	NA	8.9 (6.2)	NA
Disease stage of subtypes, No. (%)	SCD: 2 (0.8); MCI: 46 (18.8); ADD: 197 (80.4)	bvFTD: 32 (41.6); svPPA: 29 (37.7); nfvPPA: 11 (14.3); CBS or PSPS: 5 (6.4)	NA
Aβ-PET positivity, No. (%)	245 (100)	7 (9.1)	<.001
Plasma biomarker level, pg/mL			
p-tau217	7.7 (4.1)	3.4 (2.4)	<.001
GFAP	202.0 (88.8)	127.0 (78.6)	<.001
NfL	29.7 (20.2)	57.4 (43.1)	<.001

Abbreviations: Aβ, β-amyloid; ADD, Alzheimer disease dementia; *APOE*, apolipoprotein E; bvFTD, behavioral variant FTD; CBS, corticobasal syndrome; CDR-SB, Clinical Dementia Rating–Sum of Boxes; EOAD, early-onset Alzheimer disease; FTD, frontotemporal dementia; FTLD, frontotemporal lobar degeneration; GFAP, glial fibrillary acidic protein; MCI, mild cognitive impairment; MMSE, Mini-Mental State Examination; NA, nonapplicable; NfL, neurofilament light chain; nfvPPA, nonfluent/agrammatic variant primary progressive aphasia; p-tau217, phosphorylated tau 217; PET, positron emission tomography; PSPS, progressive supranuclear palsy syndrome; SCD, subjective cognitive decline; svPPA, semantic variant primary progressive aphasia.

### Association of Baseline Plasma Biomarker With Clinical Outcomes

**Figure 1.**
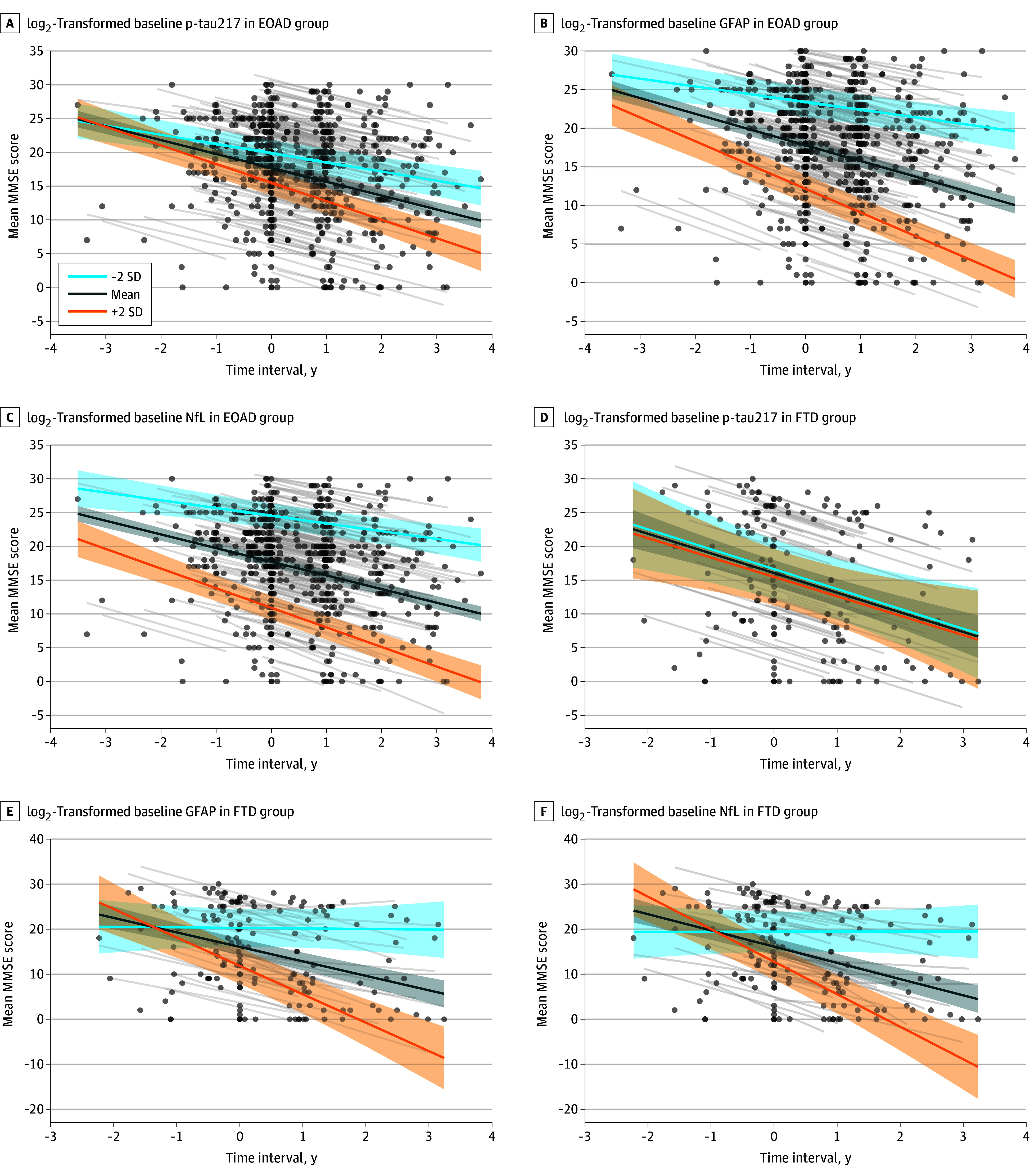
Trajectory Plot of Mini-Mental State Examination (MMSE) Scores According to Plasma Biomarker Levels Estimated MMSE trajectories with 95% CIs are shown for baseline log_2_-transformed plasma phosphorylated tau 217 (p-tau217) (A, D), log_2_-transformed glial fibrillary acidic protein (GFAP) (B, E), and log_2_-transformed neurofilament light chain (NfL) (C, F), stratified by early-onset Alzheimer disease (EOAD) and frontotemporal dementia (FTD) diagnosis groups. Biomarker × time interactions were statistically significant for all biomarkers (all *P* < .01), except for log_2_-transformed p-tau217 in the FTD group (*P* = .87). Circles represent individual observed values, and thin gray lines indicate individual patient-level estimated trajectories. Thick lines represent low (−2 SD), mean, and high (+2 SD) biomarker levels (pg/mL) within each group.

In the EOAD group, log_2_-transformed baseline p-tau217, GFAP, and NfL levels were each associated with faster decline in the MMSE score (estimate [SE], –0.390 [0.127], *P* = .002; –0.775 [0.164], *P* < .001; –0.679 [0.182], *P* < .001, respectively) (Figure 1) and the CDR-SB score (0.401 [0.099], *P* < .001; 0.535 [0.126], *P* < .001; 0.693 [0.122], *P* < .001, respectively) (Table 2). In the EOAD group, subgroup analyses stratified by baseline CDR (<1 vs ≥1) showed distinct biomarker and time associations. In the subgroup with a CDR less than 1 (n = 91), p-tau217 and GFAP levels were associated with worsening in the MMSE score (estimate [SE], –0.388 [0.159], *P* = .02 and –0.736 [0.201], *P* < .001) and the CDR-SB score (estimate [SE], 0.443 [0.093], *P* < .001 and 0.522 [0.118], *P* < .001). NfL level was also associated with progression in the CDR-SB score (estimate [SE], 0.452 [0.150]; *P* = .003). In the subgroup with CDR 1 and higher (n = 154), p-tau217 level was not associated with longitudinal clinical outcomes (all *P* > .30), whereas GFAP level was associated with decline in the MMSE score (estimate [SE], −0.561 [0.252]; *P* = .03) and NfL level was associated with progression in the CDR-SB score (estimate [SE], 0.519 [0.189]; *P* = .006) (eTable 2 in Supplement 1).

**Table 2.** Association Between Baseline Plasma Biomarker Levels and Cognitive and Functional Decline

Outcome and fixed effects^a^	Estimate (SE)	Standardized β coefficient (SE)	*P* value
**EOAD group**			
MMSE score			
Log_2_-transformed p-tau217 × time	−0.390 (0.127)	−0.055 (0.018)	.002
Log_2_-transformed GFAP × time	−0.775 (0.164)	−0.083 (0.018)	<.001
Log_2_-transformed NfL × time	−0.679 (0.182)	−0.071 (0.019)	<.001
**CDR-SB score**			
Log_2_-transformed p-tau217 × time	0.401 (0.099)	0.083 (0.021)	<.001
Log_2_-transformed GFAP × time	0.535 (0.126)	0.085 (0.020)	<.001
Log_2_-transformed NfL × time	0.693 (0.122)	0.108 (0.019)	<.001
**FTD group**			
MMSE score			
Log_2_-transformed p-tau217 × time	0.071 (0.418)	0.009 (0.051)	.87
Log_2_-transformed GFAP × time	−2.118 (0.566)	−0.179 (0.046)	<.001
Log_2_-transformed NfL × time	−2.360 (0.428)	−0.259 (0.044)	<.001
FTLD-modified CDR-SB score			
Log_2_-transformed p-tau217 × time	−0.103 (0.374)	−0.015 (0.055)	.78
Log_2_-transformed GFAP × time	0.167 (0.419)	0.017 (0.042)	.69
Log_2_-transformed NfL × time	0.766 (0.436)	0.097 (0.055)	.08

Abbreviations: CDR-SB, Clinical Dementia Rating–Sum of Boxes; EOAD, early-onset Alzheimer disease continuum; FTD, frontotemporal dementia; FTLD, frontotemporal lobar degeneration; GFAP, glial fibrillary acidic protein; MMSE, Mini-Mental State Examination; NfL, neurofilament light chain; p-tau217, phosphorylated tau 217.

^a^ All plasma biomarker levels were log_2_-transformed to reduce skewness. Models were adjusted for age, sex, and educational level, with additional adjustment for apolipoprotein E ε4 carrier status in the EOAD group only.

In the FTD group, GFAP and NfL levels were associated with MMSE score decline (estimate [SE], –2.118 [0.566], *P* < .001 and –2.360 [0.428], *P* < .001), whereas p-tau217 level had no such association (estimate [SE], 0.071 [0.418]; *P* = .87) (Figure 1). However, no biomarker was significantly associated with change in the FTLD-modified CDR-SB score (estimate [SE], p-tau217: –0.103 [0.374], *P* = .78; GFAP: 0.167 [0.419], *P* = .69; NfL: 0.766 [0.436], *P* = .08) (Table 2). In FTD, subgroup analyses were conducted among participants with Aβ-negative results (n = 60) and those younger than 70 years (n = 61) (eTable 3 in Supplement 1). Results were largely consistent with the primary analysis; however, the association of NfL level with worsening FTLD-modified CDR-SB score became significant in the subgroup with Aβ-negative results (estimate [SE], 1.024 [0.476]; *P* = .03). In stratified analyses by FTD subtype, behavioral variant FTD showed significant associations of GFAP (estimate [SE], −3.283 [0.804]; *P* < .001) and NfL (estimate [SE], −2.822 [0.484]; *P* < .001) with faster MMSE score decline as well as association of NfL with worsening FTLD-modified CDR-SB score (estimate [SE], 1.618 [0.646]; *P* = .02). In semantic variant primary progressive aphasia, GFAP was associated with FTLD-modified CDR-SB score worsening (estimate [SE], 1.971 [0.603]; *P* = .003) and marginally with MMSE score decline (estimate [SE], −1.569 [0.766]; *P* = .05) (eTable 4 in Supplement 1).

### Longitudinal Plasma Biomarker Changes in the EOAD and FTD Groups

**Figure 2.**
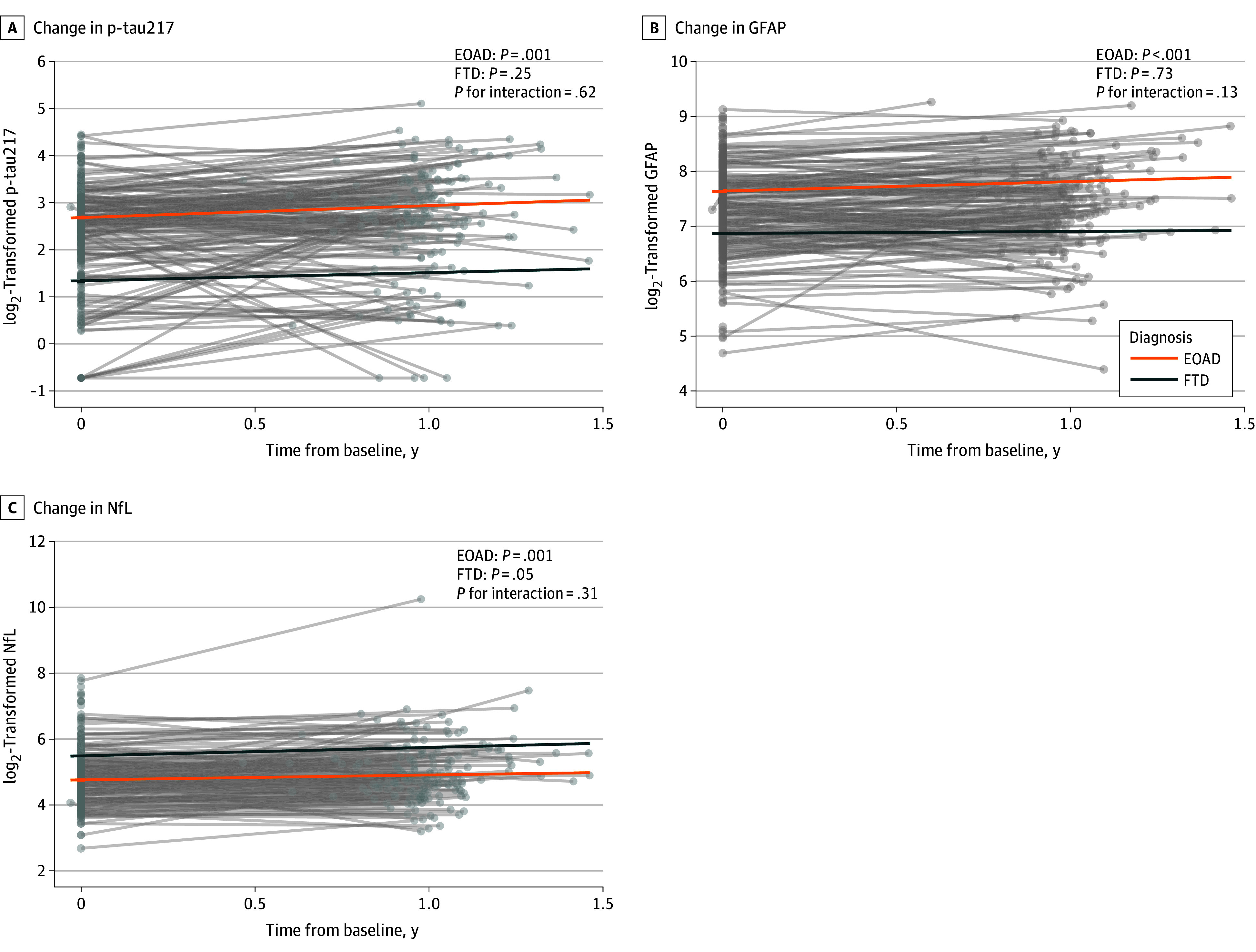
Trajectory Plot of the Longitudinal Biomarker Level Changes in the Early-Onset Alzheimer Disease (EOAD) and Frontotemporal Dementia (FTD) Diagnosis Groups *P* values were derived from the fixed effect of time interval in linear mixed-effects models that tested whether plasma biomarker trajectories change over time, adjusted for age and sex within each group (with additional adjustment for apolipoprotein E ε4 in the EOAD models). *P* values for interaction were derived from the fixed effect of the time × diagnosis term in the linear mixed-effects model, adjusted for age and sex in the combined cohort. Error bars represent 95% CIs. Circles represent individual observed values, and thin gray lines indicate individual observed trajectories. GFAP indicates glial fibrillary acidic protein; NfL, neurofilament light chain; p-tau217, phosphorylated tau 217.

In the EOAD group, all log_2_-transformed biomarker levels increased significantly over time (p-tau217: 0.253 [0.077] pg/mL, *P* = .001; GFAP: 0.173 [0.040] pg/mL, *P* < .001; NfL: 0.149 [0.045] pg/mL, *P* = .001). In the FTD group, only the NfL level showed a pattern of increase (0.251 [0.127] pg/mL; *P* = .05), but this did not reach statistical significance. Both p-tau217 (0.180 [0.155] pg/mL; *P* = .25) and GFAP (0.033 [0.094] pg/mL; *P* = .73) levels showed no significant change (Figure 2; eTable 5 in Supplement 1).

### Association of Plasma Biomarker Changes With Clinical Outcomes

In the EOAD group, annualized increases in GFAP and NfL levels were associated with faster MMSE score decline (estimate [SE], –0.005 [0.002], *P* = .007 and –0.010 [0.003], *P* = .001), whereas annualized p-tau217 level change was not (estimate [SE], 0.002 [0.032]; *P* = .96). For functional progression, only annualized p-tau217 level change was significantly associated with CDR-SB score increase (estimate [SE], 0.072 [0.024]; *P* = .003), while annualized GFAP (estimate [SE], 0.002 [0.001]; *P* = .11) and NfL (estimate [SE], 0.002 [0.001]; *P* = .06) level changes were not associated with CDR-SB score increase. In the FTD group, none of the annualized changes in plasma biomarker levels were significantly associated with changes in the MMSE and FTLD-modified CDR-SB scores (Table 3). When plasma biomarkers were log_2_-transformed, only annualized changes in NfL level were significantly associated with MMSE score decline in the EOAD group (estimate [SE], –0.544 [0.255]; *P* = .03) (eTable 6 in Supplement 1).

**Table 3.** Association Between Plasma Biomarker Level Changes and Cognitive and Functional Decline

Outcome and fixed effects^a^	Estimate (SE)	Standardized β coefficient (SE)	*P* value
**EOAD group**			
MMSE score			
Annualized p-tau217 × time	0.002 (0.032)	0.001 (0.022)	.96
Annualized GFAP × time	−0.005 (0.002)	−0.060 (0.022)	.007
Annualized NfL × time	−0.010 (0.003)	−0.154 (0.045)	.001
CDR-SB score			
Annualized p-tau217 × time	0.072 (0.024)	0.072 (0.024)	.003
Annualized GFAP × time	0.002 (0.001)	0.037 (0.023)	.11
Annualized NfL × time	0.002 (0.001)	0.054 (0.029)	.06
**FTD group**			
MMSE score			
Annualized p-tau217 × time	0.295 (0.218)	0.079 (0.058)	.18
Annualized GFAP × time	−0.003 (0.014)	−0.016 (0.075)	.84
Annualized NfL × time	−0.020 (0.029)	−0.061 (0.090)	.50
FTLD-modified CDR-SB score			
Annualized p-tau217 × time	0.150 (0.286)	0.036 (0.069)	.60
Annualized GFAP × time	0.008 (0.010)	0.043 (0.058)	.46
Annualized NfL × time	0.028 (0.019)	0.090 (0.061)	.15

Abbreviations: CDR-SB, Clinical Dementia Rating–Sum of Boxes; EOAD, early-onset Alzheimer disease continuum; FTD, frontotemporal dementia; FTLD, frontotemporal lobar degeneration; GFAP, glial fibrillary acidic protein; MMSE, Mini-Mental State Examination; NfL, neurofilament light chain; p-tau217, phosphorylated tau 217.

^a^ The annualized biomarker variable indicates the annualized change in biomarker level, calculated as change in biomarker divided by change in time on the raw scale (pg/mL per year). Models were adjusted for baseline biomarker level, age, sex, and educational level, with additional adjustment for apolipoprotein E ε4 carrier status in the EOAD group only.

### Sensitivity Analyses Excluding Genetic Cases

Two patients carrying the presenilin 1 (*PSEN1*) variant and 5 patients with the annexin A11 (*ANXA11*) variant were included in the EOAD and FTD groups, respectively. In the FTD group, sporadic cases had worse cognition than the genetic cases (median [IQR] score, MMSE: 17.0 [10.0-24.0] vs 26.0 [26.0-27.0], *P* = .005; CDR-SB: 5.00 [3.0-10.9] vs 1.50 [0.50-2.00], *P* = .003) and had higher mean (SD) NfL level (59.3 [43.8] pg/mL vs 29.6 [8.8] pg/mL; *P* < .001). Excluding genetic cases did not change the main findings in analyses (eTables 7-10 in Supplement 1).

## Discussion

The present study aimed to investigate the longitudinal changes in plasma p-tau217, GFAP, and NfL concentration, particularly in the EOAD and FTD groups, and their association with clinical progression. Our results highlight disease-specific plasma biomarker dynamics and their potential utility in monitoring disease progression in early-onset dementia.

Our first major finding was that high baseline p-tau217, GFAP, and NfL levels were significantly associated with all clinical outcomes in the EOAD group. In contrast, in the FTD group, only GFAP and NfL levels were associated with MMSE score decline. Our findings in EOAD are consistent with those in previous studies.^12,29,30,31,32,33^ The association of p-tau217 and GFAP levels with clinical outcomes was of greater magnitude at earlier stages (CDR <1), whereas p-tau217 level had no such association at later stages (CDR ≥1). This finding suggests that plasma biomarkers, particularly p-tau217, may be informative in earlier stages of EOAD. The associations of GFAP and NfL concentrations with MMSE score decline in the FTD group are partially consistent with results of earlier studies,^34,35^ suggesting their potential as prognostic markers in FTD. NfL level was also associated with FTLD-modified CDR-SB score worsening in Aβ-negative FTD, suggesting pronounced clinical relevance of NfL in pure FTD. However, GFAP level was not associated with FTLD-modified CDR-SB score, and the associations between plasma biomarker levels and the FTLD-modified CDR-SB score in the total FTD group were of lesser magnitude than those observed with the MMSE score. This finding may be attributed to the limited follow-up assessments using the FTLD-modified CDR-SB. Alternatively, the heterogeneity of FTD syndromes may have contributed to these findings.^36,37^ Specifically, FTD syndromes exhibit varying rates of cognitive and functional decline,^38,39^ and the FTLD-CDR scale—while generally more sensitive than the conventional CDR^40^—may not demonstrate equivalent sensitivity to clinical progression across different FTD subtypes. In addition, the FTLD-CDR scale may not be able to fully capture the multidomain deficits, including motor dysfunction particularly prominent in corticobasal syndrome or progressive supranuclear palsy syndrome,^41^ which may limit its consistency in capturing disease progression in FTD. In addition, the heterogeneous pathologies of FTD subtypes may underlie the inconsistent associations with plasma biomarkers, consistent with prior studies showing variable NfL and GFAP elevations across FTD subtypes depending on the underlying pathologies or causative sequence variant.^42,43,44^ Considering the clinical and biological heterogeneity within FTD,^36^ our exploratory analyses by FTD subtypes (eTable 4 in Supplement 1) showed that in behavioral variant FTD, both GFAP and NfL levels were associated with cognitive decline, whereas in semantic variant primary progressive aphasia, only GFAP level was associated with cognitive decline. However, these findings warrant cautious interpretation given the reliance on clinical diagnoses, small subgroup sizes, and limited follow-up. Accordingly, larger studies with pathological subtyping of FTD syndromes are needed to clarify the roles of plasma GFAP and NfL in FTD.

Our second finding was that plasma biomarkers followed distinct longitudinal trajectories in EOAD and FTD. In the EOAD group, the levels of all 3 biomarkers increased significantly over time, consistent with previous findings of Aβ-dependent increases in p-tau217 level in preclinical and symptomatic AD^30,31^ and of longitudinal increases in NfL and GFAP levels in symptomatic AD.^33,45^ In contrast, in the FTD group, only NfL level showed a pattern of increase, which is in line with a recent study reporting that only NfL, not GFAP, level showed significant longitudinal increases in FTD,^46^ supporting the notion that NfL outperforms GFAP in the diagnosis and monitoring of FTD. Although GFAP, a well-established nonspecific marker of astrocytic activation, is known to be elevated in FTD in several studies,^47,48,49^ its diagnostic accuracy for FTD has been inconsistent.^50,51^ Only a few studies have reported that blood GFAP levels can distinguish individuals with FTD from controls, including psychiatric disorders.^34^ Thus, relatively greater elevation of GFAP level in AD compared with FTD in the present study may suggest that GFAP has a more robust association with Aβ pathology than with heterogeneous FTLD pathology.^49,52,53^

Our third finding was that annualized changes in levels of all 3 biomarkers showed outcome-specific associations with clinical decline (GFAP and NfL levels with MMSE score; p-tau217 level with CDR-SB score) in EOAD. In contrast, none of the biomarker changes were linked to both FTLD-modified CDR-SB and MMSE score changes in FTD. The lack of association between p-tau217 level change and MMSE score change contrasts with prior studies in individuals with Aβ-positive result but without cognitive impairment and individuals with mild cognitive impairment, where increases in p-tau217 level were associated with cognitive decline.^31,54,55^ Age is unlikely to account for this discrepancy, as a previous study demonstrated significant associations between p-tau217 level and cognitive decline in individuals with preclinical AD with a mean (SD) age of 62.9 (6.06) years.^55^ Rather, a more plausible explanation may lie in the clinical composition of our cohort: more than 80% of participants had AD dementia, and their p-tau217 levels may already be markedly elevated and have plateaued.^56^ Concurrent floor or ceiling effects in the MMSE may further limit the ability of biomarker changes to track cognitive decline.^57^ By contrast, the CDR-SB might be able to capture broader cognitive and functional decline across a wider severity range,^57^ explaining the discrepancy between CDR-SB and MMSE findings. Given that p-tau217 reflects Aβ-associated tau phosphorylation early in the disease course,^54^ it may be less sensitive to later, dynamic progression of tau pathology. In this context, downstream markers, such as GFAP and NfL, may better track ongoing neurodegeneration and therefore show associations with MMSE score decline in advanced disease. In the FTD group, we did not observe associations between NfL level changes and clinical outcomes, which is inconsistent with previous studies including individuals carrying presymptomatic FTD sequence variant, suggesting that NfL level changes are associated with phenoconversion and cognitive decline in FTD.^10,58,59^ This discrepancy may be attributed to different clinical stage^58^ and limited statistical power due to the small sample. Future studies with larger symptomatic FTD cohorts with systematic subtype classification are warranted to validate these findings.

### Strengths and Limitations

This study has several strengths, including well-phenotyped participants with EOAD and FTD and a well-structured follow-up of plasma biomarkers and clinical outcomes. In particular, studies that have investigated longitudinal changes of plasma GFAP levels in EOAD samples, as in this study, are rare.

Several limitations of this study should be acknowledged. First, the absence of a normal cognition control group limits our ability to understand the magnitude of plasma biomarker changes in each disease subtype. Second, comparison with late-onset AD is warranted to elucidate the potential distinction from EOAD regarding the prognostic utility of plasma biomarkers. Third, the number of patients with FTD in this cohort was relatively small, resulting in limited statistical significance. Fourth, the finding that patients lost to follow-up had worse baseline cognition scores and higher biomarker levels suggests that informative censoring or selection bias may have attenuated the estimated associations between biomarkers and outcomes. Fifth, stage-specific analyses in the EOAD group and subtype-specific analyses in the FTD group were limited. Finally, the follow-up period was short. To address these limitations, the ongoing LEAF2 phase is enrolling additional patients and controls with normal cognition, has an extended follow-up period, and is planning comparative studies involving cohorts with late-onset AD.

## Conclusions

In this multicenter, prospective cohort study of patients with EOAD and FTD, the clinical relevance of plasma biomarker levels and longitudinal changes may vary between EOAD and FTD. These findings may inform future clinical practice and trial design regarding stratifying patient populations and monitoring clinical progression, particularly in EOAD. Further research is warranted to identify novel biomarkers and outcome measures for FTD.

## Notes

Supplement 1.
eMethods
**eTable 1.** Comparison of Baseline Characteristics Between Participants With and Without Follow-up **eTable 2.** Association Between Baseline Plasma Biomarkers and Cognitive and Functional Decline According to Baseline CDR in EOAD **eTable 3.** Association Between Baseline Plasma Biomarkers and Cognitive and Functional Decline in FTD subgroups: Aβ-Negative FTD and FTD Aged <70 Years **eTable 4.** Association Between Baseline Plasma Biomarkers and Cognitive and Functional Decline by FTD Subtypes **eTable 5.** Plasma Biomarker Trajectories in EOAD and FTD **eTable 6.** Association Between Plasma Biomarker Changes and Cognitive and Functional Decline **eTable 7.** Comparison of Clinical Characteristics Between Genetic vs. Non-genetic Cases **eTable 8.** Association Between Baseline Plasma Biomarkers and Cognitive and Functional Decline in Non-genetic Cases **eTable 9.** Longitudinal Plasma Biomarker Changes Over Time in Genetic and Non-genetic Cases **eTable 10.** Association Between Plasma Biomarker Changes and Cognitive and Functional Decline in Non-genetic Cases
eReferences

Supplement 2.
Nonauthor Collaborators

Supplement 3.
Data Sharing Statement
