# Supplementary material for: Plasma Biomarkers and Clinical Outcomes in Early-Onset Dementia
Source: JAMA Netw Open. 2026 Apr 29;9(4):e269687. doi: 10.1001/jamanetworkopen.2026.9687 (PMC13129882; doi:10.1001/jamanetworkopen.2026.9687)
Supplement: Supplement 1. — eMethods eTable 1. Comparison of Baseline Characteristics Between Participants With and Without Follow-up eTable 2. Association Between Baseline Plasma Biomarkers and Cognitive and Functional Decline According to Baseline CDR in EOAD eTable 3. Association Between Baseline Plasma Biomarkers and Cognitive and Functional Decline in FTD subgroups: Aβ-Negative FTD and FTD Aged <70 Years eTable 4. Association Between Baseline Plasma Biomarkers and Cognitive and Functional Decline by FTD Subtypes eTable 5. Plasma Biomarker Trajectories in EOAD and FTD eTable 6. Association Between Plasma Biomarker Changes and Cognitive and Functional Decline eTable 7. Comparison of Clinical Characteristics Between Genetic vs. Non-genetic Cases eTable 8. Association Between Baseline Plasma Biomarkers and Cognitive and Functional Decline in Non-genetic Cases eTable 9. Longitudinal Plasma Biomarker Changes Over Time in Genetic and Non-genetic Cases eTable 10. Association Between Plasma Biomarker Changes and Cognitive and Functional Decline in Non-genetic Cases eReferences [file jamanetwopen-e269687-s001.pdf]

Supplemental Online Content

Jang H, Lee SM, Kim HJ, et al; Longitudinal Study of Early-onset Dementia and Family Members (LEAF) Investigators. Plasma biomarkers and clinical outcomes in early-onset dementia. *JAMA Netw Open*. 2026;9(4):e269687. doi:10.1001/jamanetworkopen.2026.9687

eMethods

eTable 1. Comparison of Baseline Characteristics Between Participants With and Without Follow-up

eTable 2. Association Between Baseline Plasma Biomarkers and Cognitive and Functional Decline According to Baseline CDR in EOAD

eTable 3. Association Between Baseline Plasma Biomarkers and Cognitive and Functional Decline in FTD subgroups: Aβ-Negative FTD and FTD Aged <70 Years

eTable 4. Association Between Baseline Plasma Biomarkers and Cognitive and Functional Decline by FTD Subtypes

eTable 5. Plasma Biomarker Trajectories in EOAD and FTD

eTable 6. Association Between Plasma Biomarker Changes and Cognitive and Functional Decline

eTable 7. Comparison of Clinical Characteristics Between Genetic vs. Non-genetic Cases

eTable 8. Association Between Baseline Plasma Biomarkers and Cognitive and Functional Decline in Non-genetic Cases

eTable 9. Longitudinal Plasma Biomarker Changes Over Time in Genetic and Non-genetic Cases

eTable 10. Association Between Plasma Biomarker Changes and Cognitive and Functional Decline in Non-genetic Cases

eReferences

This supplemental material has been provided by the authors to give readers additional information about their work.

© 2026 Jang H et al. *JAMA Network Open*

eMethods

1. Protocols of Aβ PET image acquisition and definition of Aβ positivity

Participants underwent 18F-florbetaben (FBB), 18F-flutemetamol (FMM), or 18F-florapronol1,2 PET scans, following the manufacturer's protocols. Specifically, a 20-minute emission PET scan in dynamic mode (consisting of 4×5-minute frames) was performed 90 minutes after administering an average dose of 311.5 MBq for FBB or 197.7 MBq for FMM, and a 30-minute emission PET scan was acquired 30 minutes after florapronol (FC119S) administration at 370 MBq. The PET images were reconstructed in a 128×128×48 matrix with a voxel size of 2×2×3.27 mm using the ordered-subset expectation maximization algorithm (iteration = 4 and subset = 20). We defined Aβ positivity (Aβ+) as follows: visual rating score on FBB PET of 2 or 3 according to the brain Aβ plaque load scoring system,3 and positive visual interpretation of FMM or florapronol PET in any one of the five brain regions (frontal, parietal, posterior cingulate and precuneus, striatum, and lateral temporal lobes) in either hemisphere.4 Eligibility for the EOAD group required Aβ PET positivity.

2. Plasma Collection and Processing

Blood was drawn and laced in tubes containing 0.5 M ethylenediaminetetraacetic acid. The samples were centrifuged at 3000 rpm for 10 minutes, separated into 0.3 mL-vial, and then stored at -80 ºC. The plasma samples were sent from each participating center to the central lab (Seoul Clinical Laboratories) and maintained at a temperature of -80 ºC during transportation to the Department of Psychiatry and Neurochemistry at the University of Gothenburg (UGOT), where they were subsequently analyzed. Upon arrival, the samples were thawed on wet ice and centrifuged at 500×g for 5 min at 4 ºC. The in-house p-tau217 assay (UGOT p-tau217) was used on Simoa HD-X[5]. Plasma GFAP and NfL were measured using the Neurology 4-Plex E kit (Quanterix, Billerica, MA, USA). All measurements were conducted by analysts who were blinded to clinical backgrounds, and a single batch of reagents was utilized in one experimental session. The intra-assay variability for these biomarker tests was maintained below 10%.

3. Statistical analyses

Plasma biomarker values below the lower limit of quantification (LLOQ) were imputed as one-half of the lowest observed value. This applied to GFAP (N = 2, FTD) and p-tau217 (N = 19; 7 EOAD, 12 FTD). Descriptive statistics were presented as mean ± standard deviation for continuous variables and number (percentage) for categorical variables. Group comparisons for continuous variables were performed using independent two-sample t-tests, and categorical variables were compared using chi-square tests. The index date was defined as the first plasma sampling date; baseline

© 2026 Jang H et al. *JAMA Network Open*

cognitive/functional measures were the assessments closest to this date, and longitudinal outcomes were aligned to the index date using all available follow-up data. For the main analyses, plasma biomarker concentrations were log2-transformed to reduced skewness. Linear mixed effects models (LME) were used for following analyses: 1) The association between baseline biomarker levels and MMSE/CDR-SB/FTLD-CDR-SB changes were investigated using the LME, including plasma biomarkers, time, and interaction terms (biomarker × time) as fixed effects. Age, sex, education years, and APOE ε4 genotype (only for the EOAD group) were included as covariates. 2) Longitudinal plasma biomarker changes were assessed with biomarker levels as dependent variables and time interval as a fixed effect, adjusting for age, sex, and APOE ε4 genotype (only for the EOAD group) within each diagnostic group. To evaluate group differences in longitudinal trajectories, LME was applied to the combined sample, including a time × diagnostic group interaction term as a fixed effect. 3) Finally, the relationship between annualized biomarker changes (biomarker change/year) and cognitive decline was analyzed using the LME, including the interaction terms of annualized biomarker changes x time. Because absolute and proportional changes represent different effect scales, annualized biomarker change was parameterized on the raw scale (absolute change) with the log2 scale (proportional change) as sensitivity analyses. Age, sex, education years, baseline plasma biomarker levels, and APOE ε4 genotype (only for the EOAD group) were included as covariates. All models included participants as random intercepts. To facilitate comparison of effect sizes across different clinical outcomes, standardized beta coefficients were obtained using the standardize_parameters() function (parameters R package), which provides standardized fixed-effect estimates for mixed-effects models. Statistical analyses were performed using R, version 4.4.1 (R Foundation for Statistical Computing) in RStudio.

© 2026 Jang H et al. *JAMA Network Open*

eTable 1. Comparison of baseline characteristics between participants with and without follow-up

	EOAD			FTD		
	No follow-up	Follow-up	P value	No follow-up	Follow-up	P value
Age	60.9 (58.1, 65.2)	62.3 (57.9, 65.5)	0.61	63.4 (60.8, 67.7)	65.5 (60.5, 70.2)	NA
Sex						
male	18 (39%)	64 (32%)	0.39	10 (43%)	19 (35%)	0.61
female	28 (61%)	135 (68%)		13 (57%)	35 (65%)	
Disease duration	5.4 (3.5, 7.2)	4.7 (2.8, 6.5)	0.21	3.8 (2.4, 5.5)	4.1 (2.5, 6.7)	0.54
APOE ε4 carriers	25 (54%)	103 (52%)	0.88			
MMSE	16.0 (9.0, 22.0)	19.0 (16.0, 24.0)	**0.003***	20.0 (8.0, 23.0)	18.0 (12.0, 26.0)	0.49
CDR-SB	6.0 (3.5, 12.0)	5.0 (3.5, 8.0)	0.09	4.5 (3.0, 8.0)	5.0 (2.0, 11.0)	0.83
Aβ PET positivity	46 (100%)	199 (100%)		0 (0%)	7 (13%)	0.1
Plasma biomarkers						
p-tau217	9.5 (5.7, 11.8)	6.9 (5.1, 9.1)	**0.02***	2.8 (2.0, 4.0)	2.7 (1.5, 5.0)	0.71
GFAP	193.0 (168.0, 275.0)	184.0 (133.0, 242.0)	0.08	105.0 (67.7, 143.0)	115.0 (87.9, 153.0)	0.76
NfL	27.80 (22.50, 37.80)	26.50 (19.60, 33.10)	0.09	60.90 (42.10, 141.00)	40.50 (25.60, 55.60)	**0.002***

Values were represented as median (interquartile range) or numbers (percentages) as appropriate.

Abbreviations: EOAD, early-onset Alzheimer’s disease; FTD, frontotemporal dementia; APOE, Apolipoprotein E; MMSE, Mini-Mental State Examination; CDR-SB, clinical dementia rating – sum of boxes; FTLD, frontotemporal lobar degeneration; p-tau217, phosphorylated tau 217; GFAP, glial fibrillary acidic protein; NfL, neurofilament light chain; PET, positron emission tomography; N/A, non-applicable

eTable 2. Association between baseline plasma biomarkers and cognitive and functional decline according to baseline CDR in EOAD

Group	Outcome	fixed effect	Estimate	Std β	SE	P value
CDR < 1	MMSE	log_2_p-tau217* time	-0.388	-0.083	0.159	**0.02**
(N=91)		log_2_GFAP *time	-0.736	-0.128	0.201	**<0.001**
		log_2_NfL *time	-0.5	-0.069	0.29	0.09
	CDR-SB	log_2_p-tau217* time	0.443	0.217	0.093	**<0.001**
		log_2_GFAP *time	0.522	0.211	0.118	**<0.001**
		log_2_NfL *time	0.452	0.145	0.15	**0.003**
CDR ≥1	MMSE	log_2_p-tau217* time	-0.174	-0.025	0.192	0.37
(N=154)		log_2_GFAP *time	-0.561	-0.057	0.252	**0.03**
		log_2_NfL *time	-0.452	-0.049	0.252	0.07
	CDR-SB	log_2_p-tau217* time	0.147	0.03	0.151	0.33
		log_2_GFAP *time	0.226	0.031	0.207	0.27
		log_2_NfL *time	0.519	0.079	0.189	**0.006**

Abbreviations: EOAD, early-onset Alzheimer's disease continuum; FTD, frontotemporal dementia; MMSE, Mini-Mental State Examination; CDR-SB, Clinical Dementia Rating-Sum of Boxes; FTLD, Frontotemporal Lobar Degeneration; p-tau217, phosphorylated-tau 217; GFAP, glial fibrillary acidic protein; NfL, neurofilament light chain; Std β, Standardized β; SE, standard error

*all plasma biomarkers were log2-transformed.

© 2026 Jang H et al. *JAMA Network Open*

eTable 3. Association between baseline plasma biomarkers and cognitive and functional decline in FTD subgroups: Aβ-Negative FTD and FTD Aged <70 Years

Group	Outcome	fixed effect	Estimate	Std β	SE	P value
FTD (Total cohort) (N=77)	MMSE	log_2_p-tau217* time	0.071	0.009	0.418	0.87
		log_2_GFAP *time	-2.118	-0.179	0.566	**<0.001**
		log_2_NfL *time	-2.36	-0.259	0.428	**<0.001**
	FTLD-CDR-SB	log_2_p-tau217* time	-0.103	-0.015	0.374	0.78
		log_2_GFAP *time	0.167	0.017	0.419	0.69
		log_2_NfL *time	0.766	0.097	0.436	0.08
FTD (Aβ-negative) (n=70)	MMSE	log_2_p-tau217* time	0.071	0.009	0.418	0.87
		log_2_GFAP *time	-2.118	-0.179	0.566	**<0.001**
		log_2_NfL *time	-2.36	-0.259	0.428	**<0.001**
	FTLD-CDR-SB	log_2_p-tau217* time	-0.239	-0.034	0.404	0.55
		log_2_GFAP *time	0.191	0.019	0.436	0.66
		log_2_NfL *time	1.024	0.13	0.476	**0.03**
FTD (age<70) (N=61)	MMSE	log_2_p-tau217* time	0.713	0.091	0.529	0.18
		log_2_GFAP *time	-3.179	-0.264	0.699	**<0.001**
		log_2_NfL *time	-2.131	-0.251	0.464	**<0.001**
	FTLD-CDR-SB	log_2_p-tau217* time	-0.21	-0.032	0.458	0.65
		log_2_GFAP *time	-0.032	-0.003	0.484	0.95
		log_2_NfL *time	0.628	0.084	0.577	0.28

Abbreviations: FTD, frontotemporal dementia; MMSE, Mini-Mental State Examination; CDR-SB, Clinical Dementia Rating-Sum of Boxes; FTLD, Frontotemporal Lobar Degeneration; p-tau217, phosphorylated tau 217; GFAP, glial fibrillary acidic protein; NfL, neurofilament light chain; Std β, Standardized β; SE, standard error

*all plasma biomarkers were log2-transformed.

© 2026 Jang H et al. *JAMA Network Open*

eTable 4. Association between baseline plasma biomarkers and cognitive and functional decline by FTD subtypes

Group	Outcome	fixed effect	Estimate	Std β	SE	P value
bvFTD	MMSE (n=24)	log_2_p-tau217* time	0.986	0.125	0.657	0.14
		log_2_GFAP *time	-3.283	-0.253	0.804	**<0.001**
		log_2_NfL *time	-2.822	-0.385	0.484	**<0.001**
	FTLD-CDR-SB (n=20)	log_2_p-tau217* time	-0.09	-0.012	0.761	0.91
		log_2_GFAP *time	1.382	0.102	1.026	0.19
		log_2_NfL *time	1.618	0.221	0.646	**0.02**
svPPA	MMSE (n=20)	log_2_p-tau217* time	-0.463	-0.059	0.523	0.38
		log_2_GFAP *time	-1.569	-0.142	0.766	**0.05**
		log_2_NfL *time	-0.865	-0.087	0.644	0.19
	FTLD-CDR-SB (n=21)	log_2_p-tau217* time	-0.203	-0.031	0.411	0.63
		log_2_GFAP *time	1.971	0.213	0.603	**0.003**
		log_2_NfL *time	0.861	0.099	0.6	0.16
nfvPPA	MMSE (n=7)	log_2_p-tau217* time	-2.354	-0.254	1.153	0.06
		log_2_GFAP *time	-0.081	-0.006	2.753	0.98
		log_2_NfL *time	-5.952	-0.261	3.709	0.13
	FTLD-CDR-SB (n=7)	log_2_p-tau217* time	-1.026	-0.218	1.144	0.39
		log_2_GFAP *time	-0.696	-0.104	0.604	0.27
		log_2_NfL *time	-0.929	-0.086	1.788	0.61

Abbreviations: FTD, frontotemporal dementia; bvFTD, behavioral variant frontotemporal dementia; svPPA, semantic variant primary progressive aphasia; nfvPPA, nonfluent/agrammatic variant primary progressive aphasia; MMSE, Mini-Mental State Examination; CDR-SB, Clinical Dementia Rating-Sum of Boxes; FTLD, Frontotemporal Lobar Degeneration; p-tau217, phosphorylated tau 217; GFAP, glial fibrillary acidic protein; NfL, neurofilament light chain; Std β, Standardized β; SE, standard error

*all plasma biomarkers were log2-transformed.

© 2026 Jang H et al. *JAMA Network Open*

eTable 5. Plasma biomarker trajectories in EOAD and FTD

Group	Outcome	fixed effects	Estimate	SE	P value
EOAD	log_2_p-tau217	time	0.253	0.077	**0.001**
	log_2_GFAP	time	0.173	0.040	**<0.001**
	log_2_NfL	time	0.149	0.045	**0.001**
FTD	log_2_p-tau217	time	0.180	0.155	0.25
	log_2_GFAP	time	0.033	0.094	0.73
	log_2_NfL	time	0.251	0.127	0.05

Abbreviations: EOAD, early-onset Alzheimer's disease continuum; FTD, frontotemporal dementia; p-tau217, phosphorylated tau 217; GFAP, glial fibrillary acidic protein; NfL, neurofilament light chain; SE, standard error

© 2026 Jang H et al. *JAMA Network Open*

eTable 6. Association between plasma biomarker changes and cognitive and functional decline

Group	outcome	fixed effects	Estimate	Std β	SE	P value
EOAD	MMSE	annualized_p-tau217 * time	0.144	0.022	0.134	0.28
		annualized_GFAP *time	-0.314	-0.027	0.261	0.23
		annualized_NfL *time	-0.544	-0.05	0.255	**0.03***
	CDRSB	annualized_p-tau217 * time	0.079	0.017	0.098	0.42
		annualized_GFAP *time	0.027	0.003	0.185	0.89
		annualized_NfL *time	0.14	0.019	0.191	0.46
FTD	MMSE	annualized_p-tau217 * time	0.122	0.015	0.514	0.81
		annualized_GFAP *time	-0.576	-0.039	0.946	0.54
		annualized_NfL *time	-1.395	-0.118	0.929	0.14
	FTLD-CDR-SB	annualized_p-tau217 * time	0.732	0.084	0.598	0.23
		annualized_GFAP *time	0.975	0.067	0.784	0.22
		annualized_NfL *time	1.333	0.113	0.769	0.09

Abbreviations: EOAD, early-onset Alzheimer's disease continuum; FTD, frontotemporal dementia; MMSE, Mini-Mental State Examination; CDR-SB, Clinical Dementia Rating-Sum of Boxes; FTLD, Frontotemporal Lobar Degeneration; p-tau217, phosphorylated tau 217; GFAP, glial fibrillary acidic protein; NfL, neurofilament light chain Std β, Standardized β; SE, standard error

*The annualized_biomarker variable indicates the annualized change in biomarker level, calculated as Δbiomarker/Δtime on the $\log_2$-transformed scale ($\log_2$-fold change per year). Models were adjusted for baseline biomarker level, age, sex, and years of education, with additional adjustment for APOE ε4 carrier status in the EOAD group only.

© 2026 Jang H et al. *JAMA Network Open*

eTable 7. Comparison of clinical characteristics between genetic vs. non-genetic cases

		Genetic cases	Non-genetic	P value
**EOAD**		**(N=2, PSEN1)**	**(N=243)**	
	age	56.75 ± 1.83	61.83 ± 5.45	0.14
	Sex, female	0 (0)	163 (67.1)	N/A
	Disease duration	4.75 ± 0.42	5.13 ± 3.03	0.39
	MMSE	27.0 (25.5, 28.5)	19 (14.5, 23.0)	**0.06**
	CDR-SB	2.25 (1.38, 3.12)	5.00, (3.50, 9.00)	0.117
	P-tau217	11.97 ± 5.56	7.68 ± 4.08	0.47
	GFAP	194.00 ± 14.14	201.11 ± 89.28	0.61
	NfL	26.90 ± 8.77	29.70 ± 20.36	0.73
**FTD**		**(N=5, ANXA11)**	**(N=72)**	
	age	67.83 ± 8.18	64.89 ± 7.30	0.47
	Sex, female	5 (100.0)	43 (59.7)	0.19
	Disease duration	6.83 ± 1.84	4.50 ± 3.23	0.04
	MMSE	26.0 (26.0, 27.0)	17.0 (10.0, 24.0)	**0.005**
	CDR-SB	1.50 (0.50, 2.00)	5.00 (3.0, 10.9)	**0.003**
	P-tau217	3.90 ± 3.51	3.35 ± 2.35	0.75
	GFAP	140.68 ± 76.93	126.28 ± 79.19	0.70
	NfL	29.62 ± 8.75	59.29 ± 43.84	**<0.001**

Data are presented as mean ± SD for normally distributed continuous variables, median (interquartile range) for non-normally distributed variables, and n (%) for categorical variables.

P values were calculated using the Wilcoxon rank-sum test for continuous variables and Fisher's exact test for categorical variables, as appropriate. P values were not computed when a variable had no variability within a group. Due to the very small number of genetic cases (EOAD, n=2; FTD, n=5), these comparisons should be interpreted with caution and are presented for descriptive purposes.

Abbreviations: EOAD, early-onset Alzheimer's disease continuum; FTD, frontotemporal dementia; PSEN1, Presenilin 1; ANXA11, Annexin A11; MMSE, Mini-Mental State Examination; CDR-SB, Clinical Dementia Rating–Sum of Boxes; p-tau217, phosphorylated tau 217; GFAP, glial fibrillary acidic protein; NfL, neurofilament light chain; SD, standard deviation

© 2026 Jang H et al. *JAMA Network Open*

eTable 8. Association between baseline plasma biomarkers and cognitive and functional decline in non-genetic cases

Group	outcome	fixed effects	Estimate	Std β	SE	P value
EOAD	MMSE	log_2_p-tau217* time	-0.388	-0.055	0.127	0.002
		log_2_GFAP *time	-0.774	-0.084	0.164	<0.001
		log_2_NfL *time	-0.678	-0.071	0.182	<0.001
	CDRSB	log_2_p-tau217* time	0.403	0.084	0.099	<0.001
		log_2_GFAP *time	0.532	0.086	0.125	<0.001
		log_2_NfL *time	0.701	0.11	0.122	<0.001
FTD	MMSE	log_2_p-tau217* time	-0.011	-0.001	0.405	0.98
		log_2_GFAP *time	-1.981	-0.178	0.544	<0.001
		log_2_NfL *time	-1.877	-0.225	0.388	<0.001
	FTLD-CDR-SB	log_2_p-tau217* time	-0.058	-0.009	0.391	0.88
		log_2_GFAP *time	0.137	0.014	0.43	0.75
		log_2_NfL *time	0.657	0.088	0.45	0.15

Abbreviations: EOAD, early-onset Alzheimer's disease continuum; FTD, frontotemporal dementia; MMSE, Mini-Mental State Examination; CDR-SB, Clinical Dementia Rating-Sum of Boxes; FTLD, Frontotemporal Lobar Degeneration; p-tau217, phosphorylated tau 217; GFAP, glial fibrillary acidic protein; NfL, neurofilament light chain; Std β, Standardized β; SE, standard error

*all plasma biomarkers were log2-transformed.

© 2026 Jang H et al. *JAMA Network Open*

eTable 9. Longitudinal plasma biomarker changes over time in genetic and non-genetic cases

Group		outcome	fixed effects	Estimate	SE	P value
EOAD	Total cohort (N=245)	log_2_ptau217	time	0.2526	0.0773	**0.001**
		log_2_GFAP	time	0.1728	0.0404	**<0.001**
		log_2_NfL	time	0.1489	0.0447	**0.001**
	No genetic abnormalities (N=241)	log_2_ptau217	time	0.2496	0.0784	**0.002**
		log_2_GFAP	time	0.17	0.0409	**<0.001**
		log_2_NfL	time	0.149	0.0452	**0.001**
	PSEN1 carrier (N=2)	log_2_ptau217	time	0.4257	0.1152	**0.01**
		log_2_GFAP	time	0.3564	0.062	**0.001**
		log_2_NfL	time	0.1019	0.183	0.60
FTD	Total cohort (N=77)	log_2_ptau217	time	0.1796	0.1553	0.25
		log_2_GFAP	time	0.0331	0.0936	0.73
		log_2_NfL	time	0.2513	0.1266	0.05
	No genetic abnormalities (N=72)	log_2_ptau217	time	0.1416	0.1678	0.40
		log_2_GFAP	time	0.0109	0.1012	0.91
		log_2_NfL	time	0.2496	0.1361	0.07
	ANXA11 carrier (N=5)	log_2_ptau217	time	0.5944	0.1629	**0.04**
		log_2_GFAP	time	0.2408	0.1338	0.16
		log_2_NfL	time	0.2869	0.1953	0.18

Abbreviations: EOAD, early-onset Alzheimer's disease continuum; FTD, frontotemporal dementia; PSEN1, Presenilin 1; ANXA11, Annexin A11p-tau217, phosphorylated tau 217; GFAP, glial fibrillary acidic protein; NfL, neurofilament light chain; SE, standard error

Given the limited sample size in genetic subgroups (PSEN1, n=2; ANXA11, n=5), subgroup-specific mixed-effects model estimates may be unstable; thus, results are interpreted descriptively.

*all plasma biomarkers were log2-transformed.

© 2026 Jang H et al. *JAMA Network Open*

eTable 10. Association between plasma biomarkers changes and cognitive and functional decline in non-genetic cases

Group	Outcome	fixed effects	Estimate	Std β	SE	P value
EOAD	MMSE	annualized_p-tau217 * time	0.001	0.001	0.032	0.98
		annualized_GFAP *time	-0.005	-0.061	0.002	0.007
		annualized_NfL *time	-0.011	-0.156	0.003	0.001
	CDRSB	annualized_p-tau217 * time	0.072	0.072	0.024	0.003
		annualized_GFAP *time	0.002	0.037	0.001	0.11
		annualized_NfL *time	0.002	0.055	0.001	0.06
FTD	MMSE	annualized_p-tau217 * time	0.14	0.035	0.3	0.64
		annualized_GFAP *time	0.011	0.066	0.01	0.31
		annualized_NfL *time	0.025	0.086	0.02	0.21
	FTLD-CDR-SB	annualized_p-tau217 * time	0.31	0.087	0.226	0.18
		annualized_GFAP *time	-0.008	-0.045	0.014	0.59
		annualized_NfL *time	-0.017	-0.058	0.03	0.57

Abbreviations: EOAD, early-onset Alzheimer's disease continuum; FTD, frontotemporal dementia; MMSE, Mini-Mental State Examination; CDR-SB, Clinical Dementia Rating-Sum of Boxes; FTLD, Frontotemporal Lobar Degeneration; p-tau217, phosphorylated tau 217; GFAP, glial fibrillary acidic protein; NfL, neurofilament light chain; Std β, Standardized β; SE, standard error

*The annualized biomarker variable indicates the annualized change in biomarker level, calculated as Δbiomarker/Δtime on the raw scale (pg/mL per year). Models were adjusted for baseline biomarker level, age, sex, and years of education, with additional adjustment for APOE *ε*4 carrier status in the EOAD group only.

© 2026 Jang H et al. *JAMA Network Open*

eReferences

1. Byun BH, Kim BI, Park SY, et al. Head-to-head comparison of 11C-PiB and 18F-FC119S for Aβ imaging in healthy subjects, mild cognitive impairment patients, and Alzheimer's disease patients. *Medicine (Baltimore)*. Mar 2017;96(12):e6441. doi:10.1097/md.0000000000006441

2. Baik K, Jeon S, Park M, et al. Comparison Between 18F-Florapronol and 18F-Florbetaben Imaging in Patients With Cognitive Impairment. *J Clin Neurol*. 5/2023;19(3):260–269.

3. Barthel H, Gertz HJ, Dresel S, et al. Cerebral amyloid-beta PET with florbetaben (18F) in patients with Alzheimer's disease and healthy controls: a multicentre phase 2 diagnostic study. *Lancet Neurol*. May 2011;10(5):424–35. doi:10.1016/S1474-4422(11)70077-1

4. Farrar GJJoNM. Regional visual read inspection of [18F] flutemetamol brain images from end-of-life and amnestic MCI subjects. 2017;58(supplement 1):1250–1250.

5. Gonzalez-Ortiz F, Ferreira PCL, González-Escalante A, et al. A novel ultrasensitive assay for plasma p-tau217: Performance in individuals with subjective cognitive decline and early Alzheimer's disease. *Alzheimer's & dementia : the journal of the Alzheimer's Association*. Feb 2024;20(2):1239–1249. doi:10.1002/alz.13525

© 2026 Jang H et al. *JAMA Network Open*
