# Supplementary material for: Plasma Biomarkers and Clinical Outcomes in Early-Onset Dementia
Source: JAMA Netw Open. 2026 Apr 29;9(4):e269687. doi: 10.1001/jamanetworkopen.2026.9687 (PMC13129882; doi:10.1001/jamanetworkopen.2026.9687)
Supplement: Supplement 2. — Nonauthor Collaborators [file jamanetwopen-e269687-s002.pdf]

Supplemental Online Content: Nonauthor Collaborators
*First name, last name, and suffix (if applicable) are required and will appear in PubMed.

*Group Name(s): LEAF							
***First Name and Middle Initial(s)**	***Last Name**	***Suffix (eg, Jr, III)**	Academic Degrees	Institution	Location (city, state/province, country)	Role or Contribution, eg, chair, principal investigator	Group (if more than 1 Group listed in the byline) and/or Subgroup (eg, Steering Committee)
Kee Hyung	Park		MD				
Hanna	Cho		MD				
Han-Kyeol	Kim		MD				
Yeshin	Kim		MD				
Jae Won	Jang		MD				
Hee Young	Kang		MD				
Sung Hoon	Kang		MD				
Soo Jin	Yoon		MD				
Kwang Ki	Kim		MD				
Hang Rai	Kim		MD				
Sang-Myung	Cheon		MD				
Daye	Yoon		MD				
Kyung Won	Park		MD				
Eun Joo	Chung		MD				
Young Ho	Park		MD				
Jae-Sung	Lim		MD				
Sungyang	Jo		MD				
Hyemin	Jang		MD				
Geon Ha	Kim		MD				
Jee Hyang	Jeong		MD				
Seong Hye	Choi		MD				
Soo Hyun	Cho		MD				
Hyun-Soo	Kim		MD				
Seung Joo	Kim		MD				
Jay Cheol	Kwon		MD				
Ae Young	Lee		MD				
Juyoun	Lee		MD				
Kyung hun	Kang		MD				
Jaeho	Kim		MD				

Supplemental Online Content: Nonauthor Collaborators
*First name, last name, and suffix (if applicable) are required and will appear in PubMed.

***First Name and Middle Initial(s)**	***Last Name**	***Suffix (eg, Jr, III)**	Academic Degrees	Institution	Location (city, state/province, country)	Role or Contribution, eg, chair, principal investigator	Group (if more than 1 Group listed in the byline) and/or Subgroup (eg, Steering Committee)
Si Eun	Kim		MD				
Hyon-Ah	Yi		MD				
Ahro	Kim		MD				
Ji Yoon	Park		MD				
Ko Woon	Kim		MD				
Yun Su	Hwang		MD				
Min Young	Chun		MD				
Dongwon	Yang		MD				
Bora	Yoon		MD				
Yun Jeong	Hong		MD				
Young Hee	Jung		MD				
Yongsoo	Shim		MD				
Byoung Seok	Ye		MD				
Hyuk Sung	Kwon		MD				
Hee-Jin	Kim		MD				
