# Supplementary material for: Plasma Biomarkers and Clinical Outcomes in Early-Onset Dementia
Source: JAMA Netw Open. 2026 Apr 29;9(4):e269687. doi: 10.1001/jamanetworkopen.2026.9687 (PMC13129882; doi:10.1001/jamanetworkopen.2026.9687)
Supplement: Supplement 3. — Data Sharing Statement [file jamanetwopen-e269687-s003.pdf]

Data Sharing Statement

Jang. Plasma Biomarkers and Clinical Outcomes in Early-Onset Dementia. *JAMA Netw Open*. Published April 29, 2026. doi:10.1001/jamanetworkopen.2026.9687

Data
Data available: No
